# Correction: DDX43 mRNA expression and protein levels in relation to clinicopathological profile of breast cancer

**DOI:** 10.1371/journal.pone.0316591

**Published:** 2024-12-26

**Authors:** Noha N. Amer, Rabab Khairat, Amal M. Hammad, Mahmoud M. Kamel

There is an error in affiliation 4 for author Mahmoud M. Kamel. The correct affiliation 4 is: Clinical Pathology Department, National Cancer Institute, Cairo University, Cairo, Egypt.
